# Genotypic and phenotypic characterisation of three local chicken ecotypes of Ghana based on principal component analysis and body measurements

**DOI:** 10.1371/journal.pone.0308420

**Published:** 2024-08-07

**Authors:** Princess K. Botchway, Esinam N. Amuzu-Aweh, Augustine Naazie, George K. Aning, Hope R. Otsyina, Perot Saelao, Ying Wang, Huaijun Zhou, Jack C. M. Dekkers, Sue J. Lamont, Rodrigo A. Gallardo, Terra R. Kelly, David Bunn, Boniface B. Kayang

**Affiliations:** 1 Department of Animal Science, University of Ghana, Legon, Accra, Ghana; 2 USAID Feed the Future Innovation Lab for Genomics to Improve Poultry, University of California, Davis, CA, United States of America; 3 School of Veterinary Medicine, University of Ghana, Legon, Accra, Ghana; 4 Department of Animal Science, University of California, Davis, CA, United States of America; 5 Department of Animal Science, Iowa State University, Ames, IA, United States of America; 6 School of Veterinary Medicine, University of California, Davis, CA, United States of America; 7 One Health Institute, University of California, Davis, CA, United States of America; Nasarawa State University, NIGERIA

## Abstract

This study aimed to characterise three Ghanaian local chicken ecotypes, namely, Interior Savannah, Forest, and Coastal Savannah, based on morphological data and single nucleotide polymorphism (SNP) genotypes. Morphological data including body weight, shank length, body girth, back length, thigh length, beak length, comb length, and wattle length were collected from 250 local chickens. DNA isolated from blood of 1,440 local chickens was used for SNP genotyping with the Affymetrix chicken 600k SNP chip. Principal component analysis showed that Forest and Coastal Savannah birds were closely related. Generally, all three ecotypes exhibited high genetic diversity, especially birds from the Interior Savannah zone. Morphological characterisation showed that ecotype (*p = 0*.*016*) and sex (*p = 0*.*000*) had significant effects on body weight. Birds of the Interior Savannah ecotype were the heaviest (*p = 0*.*004*), with mean weights of 1.23 kg for females and 1.40 kg for males. Sex also had a strong significant effect on most of the morphological measurements, but the sex * ecotype interaction effect was not significant. Very few of the feather phenotypes previously reported to be associated with heat resistance–frizzle (2%) and naked neck (1.6%)–were found in the studied populations. It is concluded that the three local ecotypes are genetically diverse but with similar morphological features and the information provided would be useful for future selection decisions.

## Introduction

Poultry play a vital role in lower and middle-income countries (LMIC) by providing protein and vital micronutrients in the form of meat and eggs [1], with few or no religious or cultural restrictions [2]. Chickens are the leading poultry species worldwide [3] and an accessible source of income to meet various household needs, including medical care and education in rural communities. In Africa, local chickens, also referred to as village chickens, are the focus of many women and youth producers, as they are smaller in size and require very little feed and other management inputs to produce desirable outputs as compared to commercial breeds [4]. Their unique taste and high cultural value contribute to the long list of attributes that make these birds the most preferred choice for backyard production in many African countries despite their low productivity [5]. These birds are also thought to be more disease resilient and hardy in the local environment and hence, have higher survival rates compared to commercial poultry [6–8]. In Ghana, the value of local chickens to enhance nutrition and aid in poverty alleviation has been well documented [4].

Characterisation of local poultry species is limited but essential for genetic conservation [4, 9]. Unfortunately, the general trend in LMICs is to focus government programs on highly selected, higher performing, commercial breeds at the expense of local breeds, thus increasing the risk of loss of these indigenous animal genetic resources [9]. With the ever-increasing human population and, hence, rising demand for highly nutritious food, as well as the threat of emerging diseases and climate change, characterisation and conservation of animal genetic resources is critical [10]. In Ghana, information on genetic characterisation of local chicken populations across ecozones is limited. Osei-Amponsah et al. [11] characterised two (Interior savannah and Forest) of the three main local chicken ecotypes of Ghana using microsatellite markers. Kayang et al. [12] expanded the study to include the Coastal savannah ecotype, while also considering mitochondrial DNA analysis with increased sample size and further recommended the use of Single Nucleotide Polymorphisms (SNPs). As part of a larger project focused on efforts to genetically improve resistance to Newcastle disease and increase productivity among indigenous poultry in Africa (US Agency for International Development Feed the Future Innovation Lab for Genomics to Improve Poultry, http://gip.ucdavis.edu), this study employed morphological traits and SNP genotypes to characterise the three local chicken ecotypes of Ghana, namely, Interior Savannah, Forest, and Coastal Savannah ecotypes [13]. This data would reinforce baseline information on the population structure of Ghanaian local chickens and help to identify ecotypes with unique features that could be useful in the fight against Newcastle disease.

## Materials and methods

### Study location and experimental chickens

**Fig 1:**
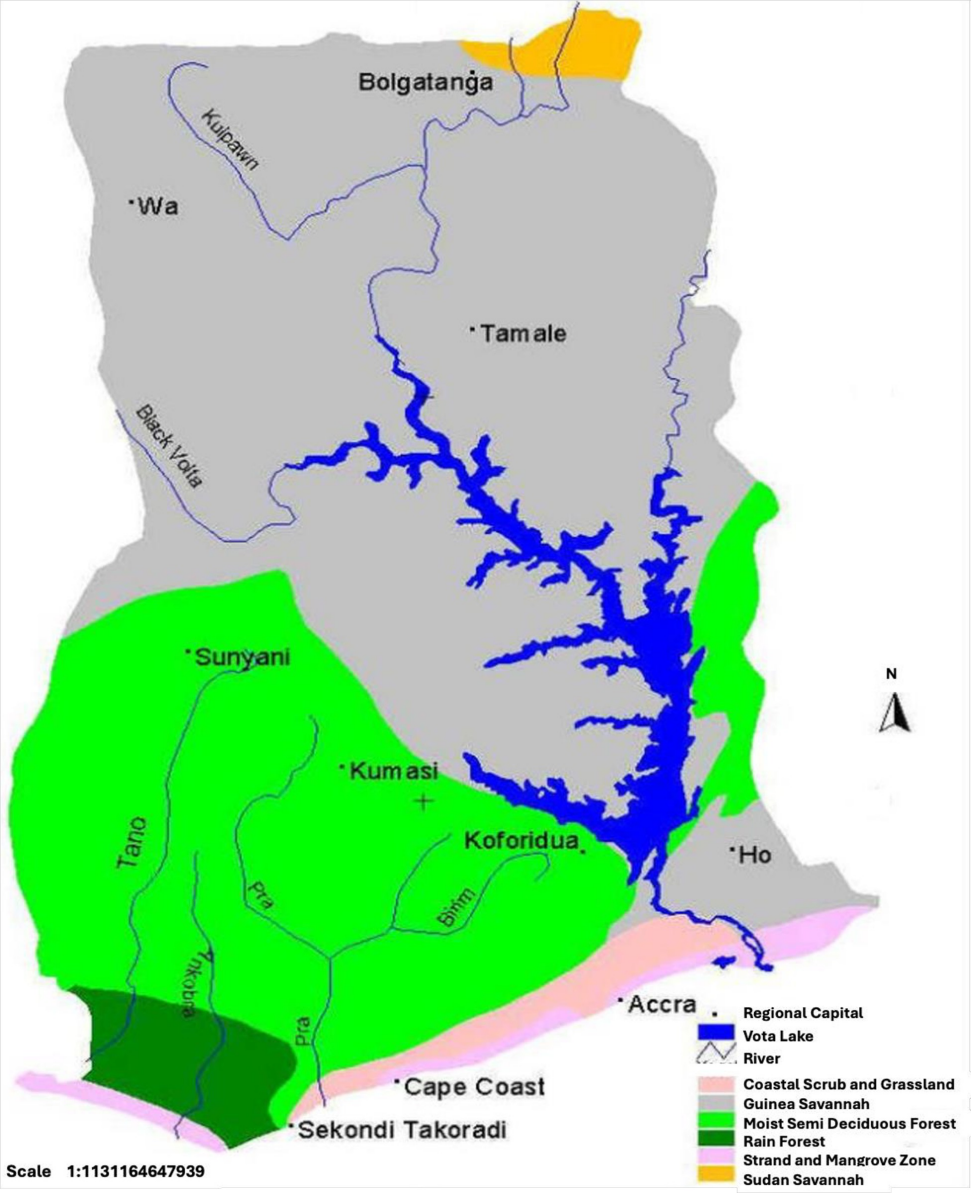
Agro-ecological zones of Ghana. Source: [15].

Local chickens were sampled from three agroecological zones of Ghana, namely, Interior Savannah (IS), which comprises the Guinea Savannah and Sudan Savannah, Forest (FO) and Coastal Savannah (CS) zones (Fig 1). These agroecological zones are described in Kayang et al. [12]. The chickens were tagged and maintained at the Livestock and Poultry Research Centre (LIPREC), University of Ghana, Legon, Accra. The study area has been described by Osei-Amponsah et al. [4]. They were grouped into 25 paternal families per ecotype, with a mating ratio of 1 male to 8 females and served as the breeding population to produce offspring used in the current study. The chicks were tagged, housed in 11 pens in a biosecure facility [14] and provided commercial Starter, Grower and Finisher feed from Agricare Feed Products Limited, Ghana. Water was provided *ad libitum*. Morphologic data were collected on 250 chickens while blood samples were collected from 1,440 chicks for genotyping and population structure analysis. All animal procedures were approved by the Council for Scientific and Industrial Research Intuitional Animal Care and Use Committee No. RPN 001/CSIR-IACUC/2014.

### Morphologic data collection

Sex, body weight and body measurements were collected on a total of 250 offspring of the breeding population (IS = 56, FO = 139 and CS = 55) at 30 weeks old, comprising 116 males and 134 females. The following linear body measurements were recorded using a measuring tape and veneer calipers (Shinwa Rules Co. Ltd., Japan, model 19971; measuring range 0–150 mm; resolution 0.01mm) where applicable: shank length, body girth, back length, thigh length, beak length, comb length, and wattle length, following the procedure described by Fayeye et al. [7]. Phenotypes associated with heat loss, including the naked neck, frizzle feather and comb type, were identified and recorded. Body weights were taken using a 5 kg (d = 5 g) Kern and Sohn GmbH (Germany) weighing scale.

### Population structure analysis

600K single nucleotide polymorphism (SNP) genotypes were available on 1,440 descendants of the breeding stock (CS = 513, FO = 520, IS = 411). SNP quality control was performed using the criteria described by Walugembe et al. [13, 16] for each ecotype. SNPs that passed quality control and that were shared by all three ecotypes were kept for further analyses. This resulted in a subset of 37K SNPs that were in near linkage equilibrium [13]. These SNPs were used in a principal component analysis (PCA) implemented with the “SNP-relate” package [17] in R version 3.3 software [18]. Multidimensional Scaling was done in Plink v1.9 software [19] and admixture analysis [13] was done using the Admixture Software [20].

### Statistical analyses

Due to unequal sample sizes in sub-groups and to account for both fixed and random effects, all the quantitative morphological traits were analysed with mixed linear models to test whether there were significant differences between ecotypes and sexes. An interaction term of ecotype*sex was also fitted. The models fitted are represented by:

$$y=X_{1}u+X_{2}b+Z_{p}p+e,$$

where y is the vector of phenotypic measurements, X_1_ is a vector of ones, *u* is the overall mean, X_2_ is the incidence matrix relating the fixed effects to vector y, b is the vector of fixed effects, Z_*p*_ is the incidence matrix relating the phenotypic observations to the vector of random pen effects, ***p***, and ***e*** is the vector of random residuals. The pens and residual effects were assumed to be independent. Fixed effects significant at *p*<0.05 were kept in the model. Significance of the random effects of pen in the models was determined by comparing likelihoods of full and reduced (excluding the pen effect) models. We then estimated marginal means per ecotype. We had very few observations for feather type (naked neck and frizzle feather phenotypes), so we did not have enough statistical power to detect any significant differences between them. All analyses were conducted using ASReml software version 4.1 [21] in R version 3.3 software [18].

## Results

### Local chicken body measurements and phenotypes

The means for body weight and the body measurements of the chickens used in the study are shown in Table 1. The Interior Savannah ecotype was the heaviest, followed by the Forest and Coastal Savannah ecotypes (Table 2). Significant differences in body measurements between ecotypes (Table 2) were found for only body weight (*p = 0*.*016*).

**Table 1:** Descriptive statistics for body weights (kg) and body measurements (cm) ± SE across three ecotypes.

	Body Weight	Back Length	Shank Length	Comb ^a^ Length	Wattle ^a^ Length	Thigh Length	Body Girth	Beak Length
**Mean**	1.23 ± 0.01	8.75 ± 0.06	3.24 ± 0.03	3.46 ± 0.05	1.63 ± 0.04	6.1 ± 0.05	9.71 ± 0.05	1.32 ± 0.01
**Range**	0.74–2.00	6.40–11.50	2.20–5.00	1.50–4.70	0.90–2.60	4.00–8.20	8.00–11.50	1.00–1.90
**Number of birds**	248	248	249	114	113	249	247	248

^**a**^ Recorded for males only.

**Table 2:** Least square means ± SE for body weights (kg) and body measurements (cm) of Ghanaian local chicken ecotypes.

Trait	Coastal Savannah	Forest	Interior Savannah	p-value
	n = 55	n = 139	n = 56	
Back Length	8.41 ± 0.14	8.80 ± 0.09	8.79 ± 0.14	*0*.*095*
Body Weight	1.20 ± 0.03^b^	1.21 ± 0.02^b^	1.30 ± 0.03^a^	*0*.*016*
Shank Length	3.22 ± 0.06	3.23 ± 0.04	3.27 ± 0.06	*0*.*748*
Comb Length^1^	3.46 ± 0.11	3.44 ± 0.07	3.55 ± 0.12	*0*.*733*
Wattle Length^1^	1.61 ± 0.08	1.63 ± 0.05	1.76 ± 0.10	*0*.*279*
Thigh Length	6.23 ± 0.10	6.09 ± 0.06	6.00 ± 0.10	*0*.*814*
Body Girth	9.80 ± 0.11	9.67 ± 0.07	9.71 ± 0.11	*0*.*471*
Beak Length	1.28 ± 0.03	1.34 ± 0.02	1.35 ± 0.03	*0*.*130*

Means in the same row with superscripts in common are not significantly different (*p>0*.*05*)

^**1**^ Recorded for males only.

Sex appeared to have a very strong effect on the body weight, thigh length, body girth, beak length, shank length, and back length differences (Table 3) among the ecotypes (*p = 0*.*00*). However, there was no significant interaction effect between ecotype and sex, for any of the traits measured (*p>0*.*05*). For all measurements, males had higher means than females (Table 3). In relation to body weight, Interior Savannah males were significantly heavier (*p<0*.*05*) than all other birds. Interior Savannah females were also the heaviest among all the females (Table 3).

**Table 3:** Least square means ± SE for body weights (kg) and body measurements (cm) of Ghanaian local chicken ecotypes by sex.

Sex	Ecotype	Trait					
		Back Length	Body Weight	Shank Length	Thigh Length	Body Girth	Beak Length
**Female**	**CS**	8.06 ± 0.18^b^	1.12 ± 0.03^c^	3.01± 0.06^b^	5.84 ± 0.21^b^	9.50 ± 0.11^b^	1.24 ± 0.02^b^
	**FO**	8.39 ± 0.14^b^	1.14 ± 0.02^c^	3.01± 0.04^b^	5.67 ± 0.17^b^	9.35 ± 0.08^b^	1.28 ± 0.02^b^
** **	**IS**	8.64 ± 0.18^b^	1.23 ± 0.03^b^	3.10 ± 0.05^b^	5.74 ± 0.22^b^	9.46 ± 0.11^b^	1.31 ± 0.02^b^
**Male**	**CS**	8.81 ± 0.18^a^	1.29 ± 0.03^b^	3.45 ± 0.06^a^	6.59 ± 0.21^a^	10.13 ± 0.11^a^	1.33 ± 0.02^a^
	**FO**	9.14 ± 0.14^a^	1.30 ± 0.02^b^	3.45 ± 0.04^a^	6.41 ± 0.18^a^	9.99 ± 0.08^a^	1.37 ± 0.02^a^
	**IS**	9.40 ± 0.19^a^	1.40 ± 0.03^a^	3.54 ± 0.06^a^	6.49 ± 0.23^a^	10.09 ± 0.11^a^	1.40 ± 0.03^a^
***P value***							
Sex		*0*.*000*	*0*.*000*	*0*.*000*	*0*.*000*	*0*.*000*	*0*.*000*
Ecotype * Sex		*> 0*.*05*	*> 0*.*05*	*> 0*.*05*	*> 0*.*05*	*> 0*.*05*	*> 0*.*05*

Means in the same column with superscripts in common are not significantly different (*p<0*.*05*)

Coastal Savannah (CS), Forest (FO) and Interior Savannah (IS)

All the birds had single combs except four male birds from the Interior Savannah ecotype that had rose combs (1.6%). Out of 250 birds used in this study, only 5 frizzle feather (2%) and 4 naked neck (1.6%; all females) phenotype birds were identified. The naked neck hens were identified in the Forest ecotype, whereas the frizzle feathered hens were identified in all ecotypes.

### Population structure

#### Principal component analysis

**Fig 2:**
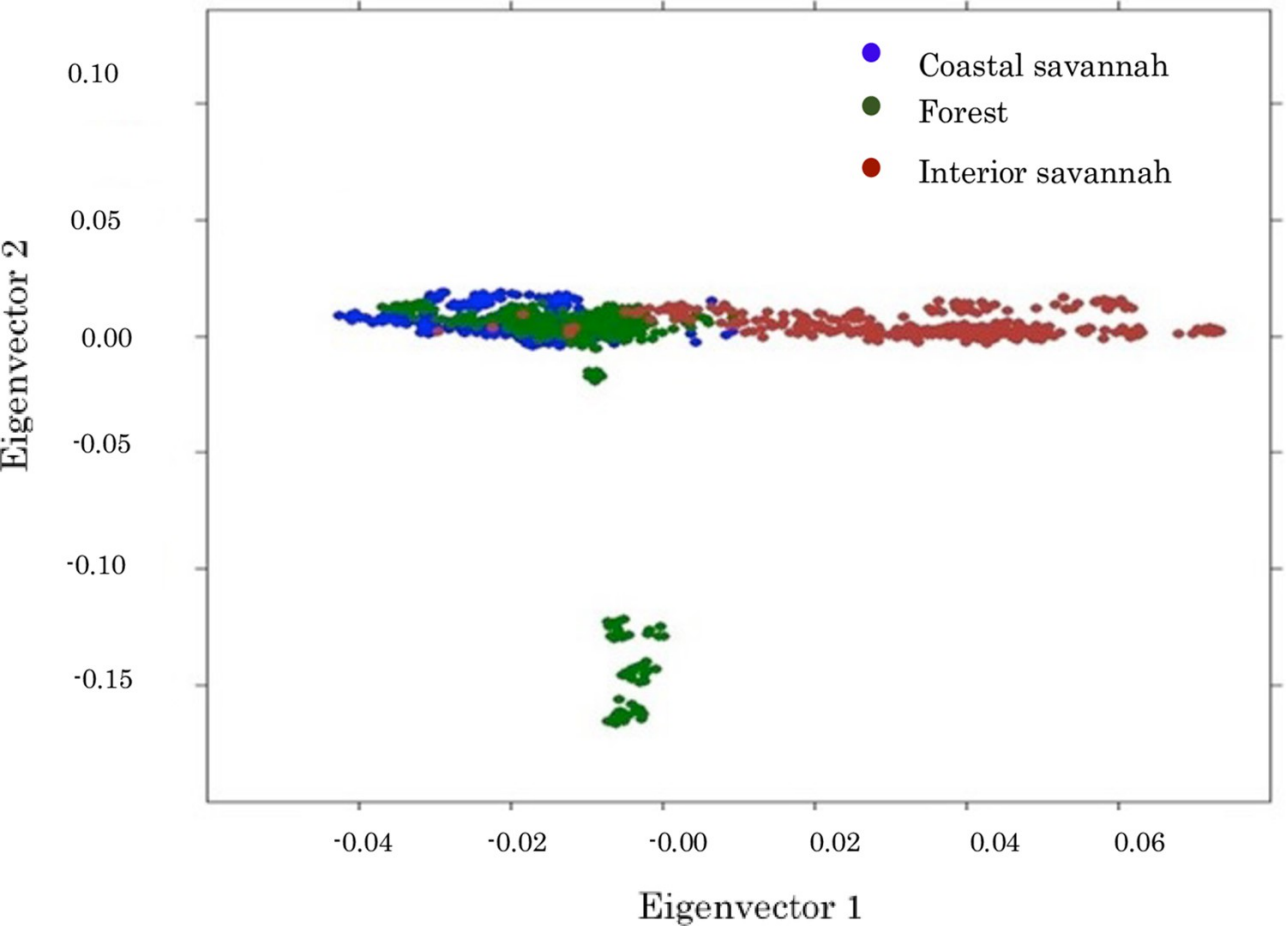
PCA plot of Ghanaian local chicken ecotypes based on 32,000 SNPs.

A plot of the first two eigenvectors based on Principal component analysis (PCA) involving 32,000 SNPs is given in Fig 2. These two eigenvectors together accounted for ~2.5% of the variation among the birds. About 3.89% of the Interior Savannah birds were found within the Forest cluster (Fig 2) while 8.46% (44 birds) of the Forest ecotype clustered closely together, separate from the main group.

#### Admixture

Admixture analyses for the three local chicken ecotypes based on identity by state, which we have previously reported in Walugembe et al [13], showed that the chickens originated from three ancestral sub-populations. Forest and Coastal Savannah populations seemed closer with a lower mean proportion of subpopulation one (0.17 and 0.20, respectively), than the Interior Savannah chickens (0.72). Further, Forest and Coastal Savannah populations had higher mean proportions of subpopulation two (0.80 and 0.69, respectively) compared to the Interior Savannah (0.02).

## Discussion

### Local chicken body measurements and phenotypes

The comparatively higher body weight for the Interior Savannah birds recorded in the present study agrees with that of Osei-Amponsah et al. [22] whose study, which was based on only the Interior Savannah and Forest ecotypes, found higher body weights for the Interior Savannah birds (1.3 to 1.8 kg) compared to the Forest ecotype (1.2 to 1.7 kg). It is probable that the local chicken farmers in the interior savannah zone have over the years selected their chickens based on weight to obtain higher market value. The mean weights reported here fall within the range of 1.3 to 1.8 kg for all the three ecotypes reported by Hagan et al. [23] and the 1.13 to 1.55 kg reported by [24] for Forest birds. The strong significant effect of sex on the body measurements including body weight was not surprising. Males have been reported to be significantly heavier than females and body weight is strongly positively correlated with these body measurements [25]. Fayeye et al. [26] in a study to compare morphometric traits between ISA Brown and Ilorin chickens in Nigeria also reported that males had higher body weights and body measurements. Similar findings were also reported by Fathi et al. [5] for Saudi native chicken breeds.

Wattles and combs play an important role in relieving heat stress and, therefore, it is not surprising that birds from the Interior Savannah zone (the zone with the hottest climate) had bigger combs and wattles. The low proportion of rose combs can be attributed to the fact that they are an adaptation to reduce heat loss from the body during very cold weather, which is not the case in the tropics. A similar trend was observed by Dahloum et al. [27] in Algerian local chicken. Notably all four birds that had rose combs in the current study were males and this agrees with Brown et al. [9], who also reported rose combs in cocks only in Tamale (in the Northern part of Ghana), which is within the Interior Savannah zone of Ghana. Although Dahloum et al. [27] reported rose combs in both sexes, the number was higher for males than for females. Other comb types, including pea and cushion, have been reported in the Interior Savannah zone and among other local chicken populations [28], but none of these were found in the current study, probably due to the different sampling methods employed in the different studies.

The proportions of different feather type reported in the current study was similar to the proportions reported for Ghanaian local chicken [29]: 2% naked neck, 2% frizzled, 96% normal. In their study of local chickens of Northern Ghana, Brown et al. [9] also reported relatively low proportions of naked neck (0.06%) and frizzle feathered birds (0.23%). Contrary to this finding, Dahloum et al. [27] reported a relatively higher percentage of naked necks (8.82%) and a lower proportion of frizzle feathered birds (0.45%) for Algerian local chickens. Although studies have shown that naked neck and frizzle feathered chickens have better production traits and are more resistant to diseases compared to normal feathered chickens, their proportions within flocks remain low. Naked neck chickens are a mutant variety reported to tolerate heat stress. The Forest zone has the highest humidity levels, ranging from 70 to 90% [23]. Chickens are homeotherms and dissipate heat generated from normal metabolic processes, while maintaining core body temperature through homeostasis [30]. While birds rely on their feather cover for insulation during cold weather, they tend to cope with hot weather by resting, drinking more water, panting, and reducing feed intake. The effectiveness of heat loss, in the quest to avoid heat stress, is hinged mainly on the humidity levels but also on minor factors such as wind, ventilation, incident sunshine, and feather cover [31]. The low proportion of frizzle birds could be attributed to the fatal effect of the gene in its homozygous form [26].

The different chicken phenotypes recorded per ecotype could be due to variant breeding objectives (other than rearing for meat and eggs) among the farmers in the three agroecological zones. Notably, the different cultural and religious uses of chickens with these peculiar chicken phenotypes per location also has an influence on selection for or against such birds for breeding. In Nigeria, Fajemilehin [32] and Yakubu [33] reported that the indigenous people sometimes referred to frizzle and naked necks as evil, sacred, or ugly birds and therefore used them for rituals, slaughtered them, or sold them at very low prices. Similar findings were reported in a survey of mutant genes in three agroecological zones of Ghana by Mensah [34]. The study also revealed that while silky feathered birds were seen as good brooders and used to hatch eggs of other birds in the Interior Savannah zone (and in some African countries), those in Bodomasi (Forest zone) were used as substitutes for sheep during rituals. Farmers in the different ecozones may therefore select their local chickens depending on these preferences and thus leading to the presence, absence, or low numbers of the various phenotypes identified in the various studies.

### Population structure analysis

Generally, the Forest and the Coastal Savannah birds seemed more closely related to each other than to the Interior Savannah birds. This finding is corroborated by the admixture analyses [13] which also shows the overlap between the Forest and the Coastal Savannah birds. This could be attributed to the proximity and easy access routes between these two agroecological zones and thus gene flow between the two zones, either through trade, rural urban drift, or interbreeding. This is in line with a similar study using microsatellite markers by Kayang et al. [12], which also showed that local chickens from the Forest and the Coastal Savannah Zones of Ghana were closely related and suggested that Savannah chickens may have derived from the Forest zone.

The isolated cluster of 44 Forest birds had a common sire and could be traced back to a farmer from Ejisu in the Ashanti Region who acquired his chickens from across the Volta Lake. The towns on the other side of the lake are close to the border which divides Ghana and Togo. It is likely, that these chickens could have been introduced into the country from neighboring Togo through trade. The wide dispersion seen in Fig 2 suggests that the local chicken population of Ghana is still genetically diverse, especially the birds from the Interior Savannah zone. The slight overlap between the Coastal Savannah and the Interior Savannah population confirms the South-North gene flow pattern postulated by Osei-Amponsah et al. [11] and corroborated by Kayang et al. [12]. The South-North gene flow could result in the loss of diversity in the local chicken genetic resource in the Interior Savannah zone over time.

## Conclusions

Results from this study indicate that the three ecotypes are similar morphologically but differ significantly in weight. Sex had a major influence on the body measurements. There is genetic variation among local chickens from the Interior Savannah zone and between the birds from the other ecozones. Although the Coastal Savannah and Forest ecotypes seemed close, they are still genetically diverse and are different from the Interior Savannah ecotype. The possible selection against certain genetic variants depending on the ecozone, as suggested in this study, and the uncontrolled South-North gene flow pattern could result in the loss of potentially valuable alleles including those associated with heat tolerance and disease resilience over time. These results could inform conservation strategies and policies as well as genetic selection decisions to boost local chicken production in Ghana.
